# Sleep in everyday life – relationship to mood and performance in young and older adults: a study protocol

**DOI:** 10.3389/fpsyg.2023.1264881

**Published:** 2023-11-23

**Authors:** Johanna Schwarz, Malin Freidle, Wessel van Leeuwen, Torbjörn Åkerstedt, Göran Kecklund

**Affiliations:** ^1^Stress Research Institute, Department of Psychology, Stockholm University, Stockholm, Sweden; ^2^Department of Clinical Neuroscience, Karolinska Institute, Stockholm, Sweden; ^3^Department of Psychology, Stockholm University, Stockholm, Sweden

**Keywords:** sleep, mood, performance, experience sampling method (ESM), intensive longitudinal, age

## Abstract

Laboratory based sleep deprivation studies demonstrate that lack of sleep impairs well-being and performance ability, but suggest that these effects are mitigated in older adults. Yet, much less is known whether day-to-day variations of sleep have similar consequences in the context of everyday life. This project uses an intensive longitudinal design to investigate the occurrence of day-to-day variations in sleep and their impact on mood and performance in everyday life and to examine whether effects differ between young and older adults. We aim to include 160 young (18–30 years) and 160 older adults (55–75 years) to complete a 21-day experience sampling method (ESM) protocol. During the ESM period, participants are asked to fill in (i) a brief morning questionnaire, (ii) 8 short daytime questionnaires addressing momentary well-being, sleepiness, stress, and mind wandering, followed by a 1 min cognitive task and (iii) a brief evening questionnaire, all delivered via a mobile phone application. Sleep will be measured using self-reports (daily questions) and objectively with wrist actigraphy. The impact of adult age on mean levels and intraindividual variability of sleep will be analyzed using mixed-effects location scale models. The impact of sleep on daily cognitive performance will be analyzed using multilevel linear mixed models. The relationship of sleep to mean values and variability of positive and negative affect in young and older adults will be analyzed using mixed-effects location scale modeling. The overarching purpose of the project is improving the current knowledge on the occurrence of day-to-day variations in sleep and their relationship to performance as well as positive and negative affect in young and older adults.

## 1 Background

Sleep is crucial for well-being, performance ability and health. A wealth of experimental sleep deprivation studies demonstrate that one night without sleep deteriorates affective functioning (Goldstein and Walker, 2014; Tomaso et al., 2021) and cognitive performance (Lim and Dinges, 2010). Surprisingly, older adults seem to be less vulnerable to the impact of sleep deprivation on mood (Schwarz et al., 2019) and cognitive performance (Philip et al., 2004; Blatter et al., 2006; Duffy et al., 2009) in controlled laboratory settings.

However, much less is known on whether similar effects occur outside the laboratory in everyday life. Experimental sleep deprivation studies are needed to test causality, but a major drawback of the rigorous approach used in these studies is the limited ecological validity. Staying awake for more than 24 h without napping or consumption of caffeine being allowed are rare occurrences in daily life for the vast majority of people. Moreover, usually only individuals that are in excellent health, and good sleepers are eligible for participation in both experimental sleep deprivation and sleep restriction studies. Yet, about 25% of the adult population in Sweden (Mallon et al., 2014) suffer from sleep problems. In particular older participants in experimental studies may hardly be representative for their age group as sleep problems increase with age (Zhang and Wing, 2006) and many health problems tend to increase with higher age. Therefore, it is essential to challenge the findings obtained in well-controlled experimental laboratory studies in “real life conditions” in order to not only better understand which consequences rather extreme forms of sleep loss in well-controlled laboratory environments may have, but which consequences the variations in sleep that individuals encounter in their daily life actually do have.

The three main goals of this project are to investigate (i) the occurrence of day-to-day variations in sleep and their impact on two main domains of everyday life, namely (ii) cognitive performance, and (iii) affect in young and older adults. To study this, we use mobile ESM including daily questionnaires and cognitive testing in combination with actigraphy to measure sleep and wakefulness objectively. In the following, the background of the three key aspects of the current project is briefly reviewed along with the respective hypotheses that were developed based upon previous research findings.

### 1.1 Association of age with mean and intraindividual variability of sleep duration, sleep efficiency, sleep timing and sleep quality

The first main aim of the study is to investigate the association of age with mean values and intraindividual variability (IIV) of sleep parameters in everyday life. It is well-known that age is an important determinant of individual differences in sleep – on average, older adults sleep shorter, more fragmented and less deep than younger adults (Ohayon et al., 2004). Moreover, sleep timing also changes with age with older adults tending to go to sleep earlier and wake earlier than young adults (Evans et al., 2021). According to a recent meta-analysis on actigraphy-assessed sleep, these shifts in sleep timing might even be a more prominent feature of adult age differences than changes in sleep duration and efficiency (Evans et al., 2021).

Yet, sleep does not only vary between individuals (i.e., interindividual differences), but also from night to night within the same person, resulting in IIV. During recent years, this IIV of sleep has been shown to predict health outcomes above and beyond mean values (Slavish et al., 2019; Chaput et al., 2020). Several factors have been associated to IIV of sleep, with age being one prominent factor (Bei et al., 2016).

A recent study using a pooled data set confirmed that sleep duration is more stable from day to day in older adults than in younger adults when measured by actigraphy or sleep diary (Messman et al., 2022). For sleep efficiency, only actigraphy data but not sleep diary data showed significant differences (Messman et al., 2022). Interestingly, those results also indicate an increase rather than a decrease in actigraphy-assessed sleep efficiency in the older age groups (Messman et al., 2022), which is at odds to a recent meta-analysis (Evans et al., 2021). To extend on previous research we will here include several sleep related outcome measures based upon on actigraphy and self-reports. This will allow a more direct comparison on how age is associated to mean and IIV of these sleep measures, as presumptive differences between outcomes and studies may in part be due to heterogeneities between studies. As discussed by Bei et al. (2016) the close association of age and IIV of sleep could in part also be associated to unexamined common causes, such as that age is associated to increased morningness. As part of the analysis strategy we plan therefore to also account for covariates such as, e.g., diurnal type, insomnia, retirement status, anxiety and depression. Moreover, differences in weekend versus weekend sleep may need to be taken into account. Generally, individuals tend to sleep longer on weekends than weekdays, but this difference consistently decreases with increasing adult age (Åkerstedt et al., 2019; Jonasdottir et al., 2021). This would imply that adult age may moderate the effect of weekend vs. weekday on IIV of sleep.

Specifically, we will investigate the following hypotheses, which are based upon previous research results:

*H1.1*: Older adults have a) a shorter mean night sleep duration and b) a lower IIV of night sleep duration than younger adults.

*H1.2*: Older adults have a) a lower mean sleep efficiency and b) a lower IIV of sleep efficiency than younger adults.

*H1.3*: Older adults have a) earlier mean bed times and b) lower IIV of bed times than younger adults.

*H1.4*: Older adults have a) earlier mean rise times and b) lower IIV of rise times than younger adults.

*H1.5*: Older adults have a) a earlier mean mid sleep and b) lower IIV of mid sleep than younger adults.

As secondary hypotheses, we will test whether weekend vs. weekday moderates the effect of age on the outcome variables (H1.6 – H1.10).

### 1.2 Daily variations in sleep and their association to cognitive performance in young and older adults

The second main aim of this study is to investigate the effect of day-to-day variations in sleep on cognitive performance in young and older adults. Declines in cognitive performance are a hallmark of sleep loss. Experimental laboratory studies show that both total and partial sleep deprivation cause deterioration across a wide range of cognitive functions (Pilcher and Huffcutt, 1996; Lim and Dinges, 2010; Lowe et al., 2017) whereas simple attention and sustained attention are most dramatically affected (Lim and Dinges, 2010). While older adults usually have slower reaction times than younger adults when sleep is not manipulated, their performance decrements in response to sleep deprivation are less pronounced than in younger adults (Philip et al., 2004; Blatter et al., 2006; Duffy et al., 2009). However, knowledge on how day-to-day changes in sleep are related to cognitive performance is scarce. One recent study found that within-person variations in day-to-day sleep duration had very limited effects on working memory performance in older adults (Lücke et al., 2022). To the best of our knowledge, no study has yet compared young and older adults. Here we will use an established mobile version of the Digit Symbol Substitution Task implemented in the PsyMate^™^ mobile application (Verhagen et al., 2019; Daniels et al., 2020), which is considered a measure of processing speed. Performance in the Digit Symbol Substitution tasks is known to be affected by sleep loss (Van Dongen et al., 2003; Lim and Dinges, 2010; Honn et al., 2020).

While the analysis will include both between and within person effects, the hypotheses focus on the within-person effects and the cross-level interaction between adult age and the within-person effect. Building on previous sleep deprivation research, we will test the following hypotheses.

*H2.1a*: Shorter sleep duration than usual (i.e., shorter sleep duration than a person’s own average) is associated to worse cognitive performance.

*H2.1b*: This association between sleep duration and cognitive performance is less strong in older adults than in young adults.

*H2.2a*: Lower sleep efficiency than usual (i.e., lower sleep efficiency than a person’s own average) is associated to worse cognitive performance.

*H2.2b*: This association between sleep efficiency and cognitive performance is less strong in older adults than in young adults.

*H2.3a*: Poorer self-reported sleep quality than usual (i.e., poorer sleep quality than a person’s own average) is associated to worse cognitive performance.

*H2.3b*: This association between self-reported sleep quality and performance is different in older adults than in young adults.

### 1.3 Association of day-to-day changes in sleep with positive and negative affect in young and older adults

The third main aim of this project is to address the relationship between sleep and positive and negative affect. A wealth of research during recent years has shown that sleep and lack thereof is associated with changes in several aspects of affective functioning (Goldstein and Walker, 2014). A recent meta-analysis concluded that sleep deprivation decreases positive mood, and to a somewhat lesser extent increases negative mood (Tomaso et al., 2021). Yet, the effect of sleep deprivation on mood appears to be diminished in older adults (Schwarz et al., 2019). Longitudinal studies addressing the relationship between day-to-day changes in sleep and affect overall also concur that poor sleep worsens mood, and vice versa (for review Konjarski et al., 2018). Those associations are stronger if both sleep and mood are measured using self-report compared to when objective indicators of sleep are used, possibly suggesting a common method bias.

There is hardly any knowledge whether adult age moderates the relationship between sleep and affect in everyday life. To our knowledge, only three experience sampling studies have so far included older adults, confirming an association between poor sleep and poor affect in older adults (Mccrae et al., 2008; Wrzus et al., 2014; Blaxton et al., 2015). One study suggests differences between middle-aged and older adults (Blaxton et al., 2015), but no comparison to young adults was made. Wrzus et al. (2014) reported that adolescents well-being was better the longer their sleep duration was, while adults and in particular older adults benefited most from sleeping their usual sleep-length. This study used only subjective sleep reports, which are known to be biased by age (Akerstedt et al., 2016). Thus, overall, it remains unclear whether there are notable differences between young and older adults in the association between sleep and affect in everyday life.

A further important aspect that has not been directly addressed is whether day-to-day changes in sleep also impact on dynamic aspects of affect the next day. Affect is not static, but transient and varies over time. Higher variability in affect has been associated to poorer psychological health (Houben et al., 2015).

Cross-sectional analyses show that poorer sleep (Leger et al., 2019) and sleep variability (Ying et al., 2022) are associated with increased variability in positive affect. Aggregate measures of affect reactivity across 8 days have also been linked to lower sleep efficiency (Ong et al., 2013). These findings suggest that poor sleep not only is associated to poorer affect on average, but also impacts on dynamic aspects of affect in everyday life. However, this has so far only been addressed in cross-sectional analyses which do not allow for distinguishing the direction of the relationship. Therefore, it is important to extend the research and focus also on intra-individual associations (Gillett et al., 2021). Here, we will use an intensive longitudinal setup to investigate whether daily changes in sleep are related to changes in both mean levels and variability of positive and negative affect the next day.

We will also investigate whether effects differ between young and older adults, because older adult age is associated to changes of affect in daily life (Carstensen et al., 2011), lower variability in positive affect (Röcke and Brose, 2013) and smaller effects of total sleep deprivation on mood (Schwarz et al., 2019). The results will contribute to a deepened understanding of how sleep is linked to affective well-being.

Specifically, we will investigate the following hypotheses.

#### Positive affect

*H3.1a*: Shorter sleep duration than usual (i.e., shorter sleep duration than a person’s own average) is associated to (i) lower mean positive affect and (ii) higher variability of positive affect.

*H3.1b*: These associations between sleep duration and (i) mean positive affect and (ii) variability of positive affect are less pronounced in older adults than in young adults.

*H3.2a*: Lower sleep efficiency than usual is associated to (i) lower mean positive affect and (ii) higher variability of positive affect.

*H3.2b*: These associations between sleep efficiency and (i) mean positive affect and (ii) variability of positive affect are less pronounced in older adults than in young adults.

*H3.3a*: Poorer self-rated sleep quality than usual is associated to a) lower mean positive affect and b) higher variability of positive affect.

*H3.3b*: These associations between self-rated sleep quality and i) mean positive affect and (ii) variability of positive affect are different in older adults than in young adults.

#### Negative affect

*H3.4a*: Shorter sleep duration than usual (i.e shorter sleep duration than a person’s own average) is associated to a) higher mean negative affect and b) higher variability of negative affect.

*H3.4b*: These associations between sleep duration and (i) mean negative affect and (ii) variability of negative affect are less pronounced in older adults than in young adults.

*H3.5a*: Lower sleep efficiency than usual is associated to a) higher mean negative affect and b) higher variability of negative affect.

*H3.5b*: These associations between sleep efficiency and (i) mean negative affect and (ii) variability of negative affect are less pronounced in older adults than in young adults.

*H3.6a*: Poorer self-rated sleep quality than usual is associated to a) higher mean negative affect and b) higher variability of negative affect.

*H3.6b*: These associations between self-rated sleep quality and i) mean negative affect and (ii) variability of negative affect are different in older adults than in young adults.

Further research questions which were not part of the original grant application are planned to be (pre-)registered at OSF.^1^

To sum up, the main purpose of the project is to investigate how sleep varies in everyday life and how day-to-day changes in sleep are associated to performance and affect in younger and older adults. We here combine the experience sampling method (ESM) and actigraphy. ESM is a structured diary method where individuals provide information in the context of their everyday life via self-report (Verhagen et al., 2016). Typically, at several (pseudo-) random times during the day, participants are prompted via signals such as push notifications of a smartphone app to report their experiences, feelings, behaviors, mental states and their context (e.g., company, location, activity) in real-time.

In this study, in addition to self-reports, participants also perform a brief cognitive performance test. Both ESM questionnaires and the cognitive test are delivered via a mobile phone app. Main advantages of ESM include the high ecological validity as data is collected in daily life and a low risk for recall bias as information is collected in the moment (Verhagen et al., 2016). The intensive longitudinal setup allows to investigate the variability of transient states and relationships between variables within the same person. Thus, studying day-to-day variability with ESM is a powerful tool to understand the impact of sleep in everyday life.

## 2 Methods

### 2.1 Design

This is an intensive longitudinal study with a mixed design. Repeated mobile ESM and actigraphy during 21 days is the within subject factor, adult age group is the between subjective factor.

### 2.2 Participants

Individuals in the age ranges 18–30 years (young adults) and 55–75 years (older adults) are eligible for inclusion in the study. We plan to include *n* = 160 in the young adult group and *n* = 160 in the older adult group.

Participants need to have a Swedish social security number, be fluent in Swedish, be able to travel to Stockholm University twice for the briefing and debriefing meeting, have no indication of cognitive impairment (Mini Mental State Examination >24) and have a smartphone. The smartphone needs to be compatible with the mobile application used for the ESM. No one but the participant should have regular access to the smartphone. Participants should also have the possibility to wear an actigraph 24/7.

Individuals are not eligible for the study if they lack the ability or confidence to use a smartphone or if their smartphone screen has a functional damage. Individuals whose work requires wakefulness between 2:00 and 5:00 once a month or more often with the exception for on-call work, are also not eligible for the study and neither are individuals who report interest in the study but later on cannot be contacted or who are no longer interested when contacted. Individuals with health conditions and/or medical treatments that likely impact daily physical activity, sleep, psychological well-being or performance ability to a great extent are also not eligible for the study. Examples of medical conditions that may lead to exclusion if present during the past 6 months are sleep disorders and/or a score of >21 on the Insomnia Severity Index (Morin et al., 2011), self-reported depression diagnosis, anxiety diagnosis, exhaustion disorder or any other psychiatric diagnoses, fibromyalgia, autism or current cancer. Examples of chronic diseases that may lead to exclusion are Parkinson’s disease or dementia. Examples of medicaments that warrant exclusion are antidepressants, or prescribed sleep medicine taken >1/week.

Participants are economically compensated for their participation using a staggered payment scheme depending on the number of daily questionnaires they fill in. The maximum amount paid is 2,940 SEK (before tax), which requires the participant to fill in a minimum of 7 ESM questionnaires per day.

This study was approved by the Swedish Ethical Review Authority (2022-04683-02).

### 2.3 Data collection procedure and study assessments

**Figure 1:**
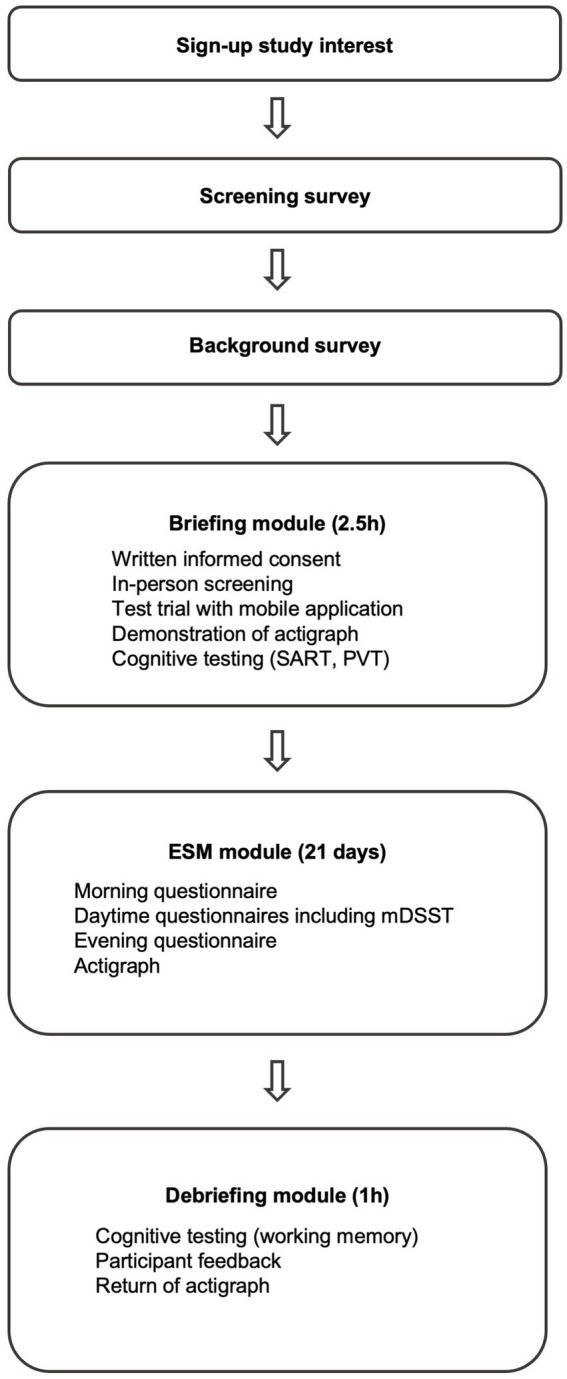
The stages of the participation process. Refer to the text for more details. ESM, Experience Sampling Method; SART, Sustained Attention to Response Task; PVT, Psychomotor Vigilance Task; mDSST, momentary Digit Symbol Substitution Task.

Participants will be recruited via advertisements in newspapers, social media, online forums, and paper flyers in the larger Stockholm area. Moreover, participants will be recruited from databases of persons who have previously participated in research projects and/or expressed interest in participation in future projects. Participants will also be invited to pass on information on the study to anyone else they know who may be interested in participating. As shown in Figure 1 the study assessments comprise the online screening and background surveys, the briefing module before the first day of ESM; the ESM module spanning 21 days; and the debriefing module after the end of the ESM module. Data collection started in September 2022.

#### 2.3.1 Screening survey

People with an interest to participate will be asked to sign up on an online form. They will then receive a link to the participant information sheet together with a link to the online screening survey via email, which will cover basic demographics (e.g., age, sex, education level) as well as the majority of the inclusion and exclusion criteria using questions regarding mobile phone use, health indicators (e.g., health problems, medication use, smoking status), sleep as well as the Insomnia Severity Index (Morin et al., 2011). Any question(s) due to ambiguous answers in the online screening form will be followed up individually by telephone. Participants that pass the online screening form will be invited to book a personal briefing meeting. They will be asked to choose the next possible time slot that is followed by 3 weeks that they expect to be representative of their usual day-to-day life.

#### 2.3.2 Background survey

Participants that pass the online screening form will also receive a link to a background online survey that includes additional background questionnaires related to sleep, health, stress, well-being, affect and cognition to further characterize the sample. Table 1 contains an overview of the elements of the screening survey, the background survey as well as the background cognitive tests.

**Table 1:** Overview of screening survey, background questionnaires and background cognitive tests.

*Demographic information*
Custom questions (screening survey)
*Sleep related questionnaires*
Insomnia severity index (Morin et al., 2011) (screening survey) Custom questions related to sleep disorders and treatment (screening survey) Epworth sleepiness scale (Johns, 1992) (background survey) Karolinska sleep questionnaire (Nordin et al., 2013) (background survey) The diurnal type scale (Torsvall and Akerstedt, 1980) (background survey) Munich chronotype questionnaire (Roenneberg et al., 2003) (background survey) Bedtime procrastination scale (Kroese et al., 2014) (background survey) Custom questions related to sleep-related behaviors (background survey)
*Health related questionnaires and measures*
Custom questions related to health (screening survey) Hospital anxiety and depression scale (Zigmond and Snaith, 1983; Lisspers et al., 1997) (background survey) Body mass index (briefing meeting)
*Stress related questionnaires*
Ford insomnia response to stress test (Drake et al., 2004) (background survey) Perceived stress scale (Cohen et al., 1983) (background survey) Shirom melamed burnout questionnaire (Melamed et al., 1992) (background survey)
*Well-being, affect and cognition related questionnaires*
Satisfaction with life scale (Diener et al., 1985) (background survey) Brief inventory of thriving, and 2 subscales of comprehensive inventory of thriving (self-worth and meaning) (Su et al., 2014) (background survey) Affective styles questionnaire (Hofmann and Kashdan, 2010) (background survey) Five facet mindfulness questionnaire (only non-reactivity subscale) (Baer et al., 2006) (background survey) Future time perspective scale (Brothers et al., 2014) (background survey) Irrational procrastination scale (Rozental et al., 2014) (background survey) Mind wandering questionnaire (Mrazek et al., 2013) (background survey) Perseverative thinking questionnaire (Ehring et al., 2011) (background survey) Positive and negative affect schedule (Watson et al., 1988) (briefing meeting)
*Cognitive tests*
Mini mental state examination (Folstein et al., 1975) (briefing meeting) Sustained attention to response task (Jackson and Balota, 2012), modified version with mind-wandering probes (briefing meeting) Psychomotor vigilance task (Khitrov et al., 2014) (briefing meeting) Working memory (symmetry span, short version) (Foster et al., 2015) (debriefing meeting) Working memory (operation span, short version) (Foster et al., 2015) (debriefing meeting)

Occasion of administration denoted in parentheses.

#### 2.3.3 Briefing module

During the personal briefing meeting, the eligibility criteria that require personal contact will be addressed: the Mini Mental State Examination (MMSE) will be administered and it will be confirmed that the participant’s phone (iPhone or Android) is compatible with the PsyMate^™^ application and that the participant is confident and able to use the application as intended.

Participants will be informed about the project in detail with the opportunity to ask questions and sign the written informed consent. The PsyMate^™^ application (see below) will be installed on the participant’s own mobile phone, and they will be instructed on how to use the application and perform a test trial for all questionnaires and the cognitive test with the possibility to ask questions. Participants will receive an actigraph to be worn on the non-dominant hand during the data collection and get instructed on their use (see section actigraphy below).

During the briefing meeting participants will also fill in the Positive and Negative Affect Schedule (Watson et al., 1988), the Karolinska Sleepiness Scale (KSS) (Åkerstedt and Gillberg, 1990) and perform two cognitive tests on the computer.

The first cognitive test performed is a modified version of the Sustained Attention to Response Task (SART) including mind wandering probes similar to Jackson and Balota (2012). Here we use a task duration of 10 min. The SART is used to measure attention, inhibition and the tendency to mind wander. In this task, one digit at a time (range 1–9) appears on the screen and the participant is instructed to respond (press a button) to all numbers except “3.” During the task the participants will be pseudo randomly interrupted with mind wandering probes. At each occasion the participant has to rate where their attention was focused on right before the probe (on task – off task) and how aware they were on where their attention was focused (aware – unaware) on 7-point Likert scales. The numbers on the Likert scale are not visible. The mind wandering probes were adapted from (Christoff et al., 2009). The main outcomes of this task will be task performance and the answers to the mind wandering probe.

Sustained attention will be measured using a 5-min long version of the PC- Psychomotor Vigilance Task (PVT) (Khitrov et al., 2014). The PC-PVT is a simple reaction time task that requires the participant to respond as fast as possible to a visual counter presented at randomly varying intervals between 2 and 5 s. Simple attention tasks such as the PVT are frequently used in sleep research (Lim and Dinges, 2010). The main outcomes of the PC-PVT will be reciprocal reaction time (RRT) and number of minor lapses (RT > 500ms).

#### 2.3.4 Experience sampling method (ESM) module

The mobile ESM data collection period will start the day after the briefing session and continue for in total 21 days. During the 3 weeks, participants are also asked to wear an actigraph. Participants are asked to live their life as normal during the data collection, and there are no requirements to follow a specific sleep schedule. About 2–3 days after the start of the mobile ESM data collection, participants will be called by phone to address possible questions regarding the data collection. Participants can also contact the research team during their data collection in case of questions.

For the mobile ESM, the PsyMate^™^ platform^2^ is being used, which consists of (i) a mobile application compatible with both iOS and Android, (ii) a cloud based data storage and (iii) a reporting tool. Participants are prompted to fill in the questionnaires via standard push notifications on their mobile phones, which will also be sent if the participant is offline. The PsyMate^™^ app also contains a note function where participants can add notes.

**Figure 2:**
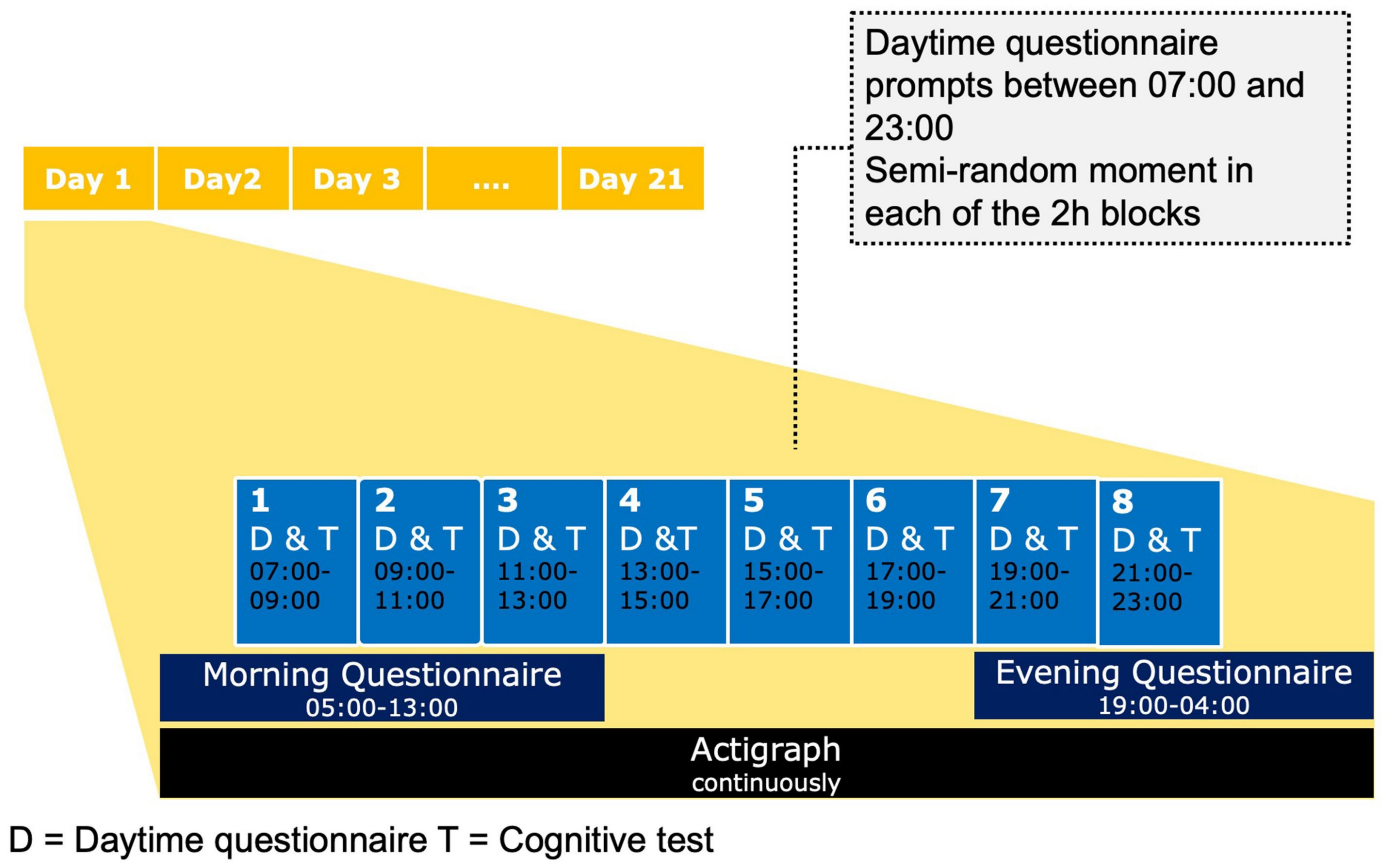
Overview of the daily schedule during the mobile ESM.

Each day, participants are asked to respond to three different types of ESM questionnaires, i.e., morning, daytime and evening questionnaires. An overview of the daily schedule during the ESM module is shown in Figure 2.

##### 2.3.4.1 Morning questionnaire

The morning questionnaires is available between 05:00 and 13:00 (interval contingent scheme). Participants are asked to fill in the questionnaire as soon as possible after getting up. The morning questionnaire consists of up to 23 items (19 standard items, 4 branched items). It includes questions adapted from the Karolinska Sleep Diary (Åkerstedt et al., 1997) focusing on sleepiness, the timing, quality and quantity of the night sleep. The items sleep quality, calmness of sleep, ease of falling asleep and sleep maintenance are used to calculate a sleep quality index with higher scores reflecting poorer sleep quality. The morning questionnaire contains also questions on sleep related behaviors (bedtime procrastination and snoozing), subjective age and expectation for the day to come.

##### 2.3.4.2 Daytime questionnaire and cognitive test

Daytime questionnaires are semi-randomly prompted 8 times a day within 120 min time blocks from 07:00 to 23:00. Prompts are delivered using standard push notification with auditory signals and a banner. Daytime prompts will expire after 20 min and are scheduled with a minimum of 30 min apart from each other. The participants are told that the prompts are available for “a short time period,” but not the exact time period.

Semi-random sampling schemes have the advantage of being unpredictable to the participants and thus minimizing reactivity to the method, while one can still assume equality for the time in between consecutive assessments, as the differences intervals are thought to equal out (Dejonckheere and Erbas, 2022).

Each daytime questionnaire contains 22 standard items and 1 branched item focusing on the momentary experience. The questionnaire includes questions on current thoughts and mind wandering, sleepiness, stress, mood, well-being, pain and situational context. Most of the items will be rated on a 7-point Likert Scale scale (1 = not at all, 4 = moderate, 7 = very). Positive affect will be measured using the items I feel happy, I feel content, I feel in good mood, which are rated on a scale from 1 to 7. Negative affect will be measured using the items I feel discouraged, I feel restless, I feel lonely, I feel peeved, which are rated on a scale from 1 to 7. Sleepiness will be measured using the Karolinska Sleepiness Scale (Åkerstedt and Gillberg, 1990), a 9-point Likert scale, with higher scores reflecting higher sleepiness. Likewise, stress will be measured using a 9-point Likert scale (Schwarz et al., 2018), with higher scores reflecting higher stress. Items on situational context have categorical response alternatives.

The daytime questionnaire will be followed by a 60s cognitive task integrated in the PsyMate^™^ mobile app, the momentary Digit Symbol Substitution Task (mDSST) (Verhagen et al., 2019; Daniels et al., 2020). The mDSST is an adaption of the Digit Symbol Substitution Task of the Wechsler Adult Intelligence Scale (Daniels et al., 2020). In the mobile version of the task the digits 1–9 are presented on top of the screen, each combined with a symbol. For each trial, a digit is presented as probe in the middle of the screen. Participants have to select the corresponding symbol on the bottom of the screen. The symbol-digit combinations are alternated for each test session, but stay the same within each test session. The main outcome measure will be number of correct trials and the secondary outcome will be percentage of correct trials. The mDSST is followed by a 7-point Likert scale question on whether the participant was distracted during the task (1 = not at all, 4 = moderate, 7 = very).

**Figure 3:**
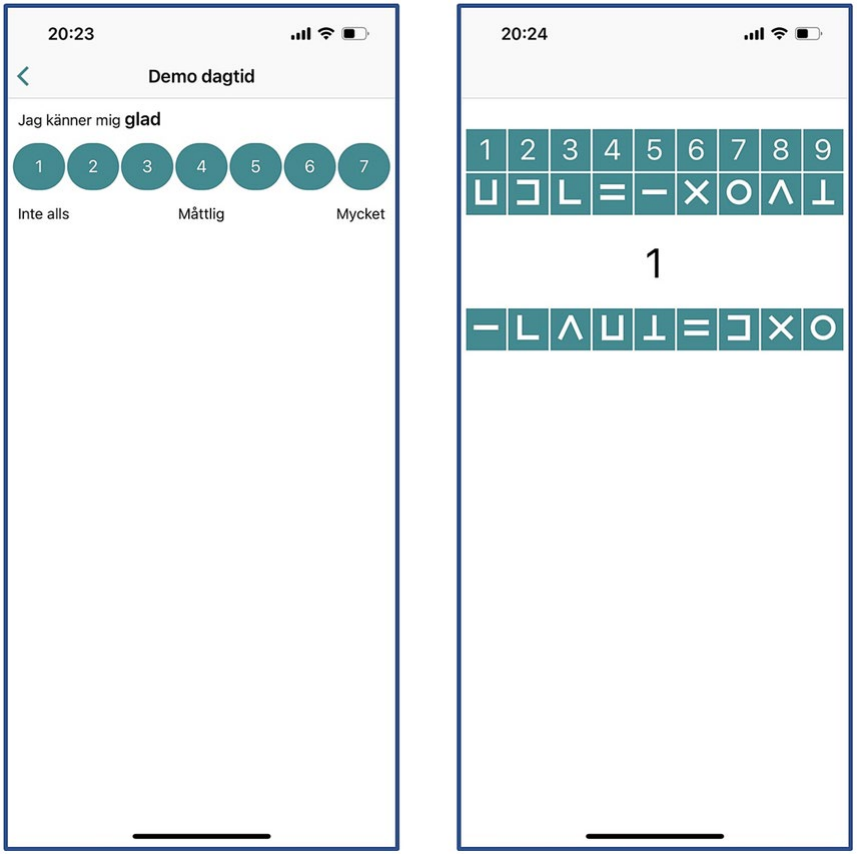
Example of a questionnaire item (left) and the momentary Digit Symbol Substitution Task (mDSST) (right) in the PsyMate^™^ application. Adapted with permission from ECS Inernational BV.

Figure 3 shows an example of an affect-related item with a 7-point Likert scale and the mDSST in the PsyMate^™^ application.

##### 2.3.4.3 Evening questionnaire

The evening questionnaire is available from 19:00 to 04:00 (interval contingent scheme). Participants are asked to fill it in right before they go to bed. The evening questionnaire consists of 20 items containing information on the current day, eudaimonic and evaluative well-being [items adapted from Diener et al. (2009) and Su et al. (2014)], subjective health and health complaints, daytime napping, occurrence of stressful and positive events [items adapted from Klaiber et al. (2021)], current sleepiness (Åkerstedt and Gillberg, 1990) and expectations for the next day. After filling in the evening questionnaire, notifications for any remaining daytime questionnaires are silent until the next morning.

##### 2.3.4.4 Actigraphy

During the 3 weeks of ESM, participants will be asked to wear an actigraph (Motionwatch 8 by CamNtech, Cambridge, United Kingdom), a watch-shaped movement monitor device on their non-dominant wrist. Participants are instructed to remove the actigraph only if they immerse the arms for a longer time in water, e.g., going swimming or taking a bath. Actigraphs measure movement along 3 axes, as well as light exposure, and actigraphy is a well-established method to measure sleep and physical activity in an objective and comfortable way (Sadeh, 2011; Falck et al., 2017). The so-called recording mode will be tri-axial mode 3 with an epoch length of 30 s.

The MotionWare software (CamNtech,Cambridge, United Kingdom) will be used to determine the actigraphy-derived sleep variables (sleep time and sleep efficiency). The auto-sleep configuration will be adjusted for start and end of the sleep based on event markers, sleep diary entries, activity level and light recordings. The settings for the software and the protocol to establish corrections for start and end of sleep will be registered at https://osf.io/8sg6v/.

#### 2.3.5 Debriefing module

After completing their respective 21 days ESM data collection, all participants have an in-person debriefing meeting at Stockholm University. During this meeting the actigraphs will be returned and two computerized working memory tasks [Symmetry Span task, Operation Span task (Foster et al., 2015)] will be carried out. During the debriefing meeting, the participants will also receive login credentials for a web-based reporting page to see a subset of their data.

### 2.4 Data analysis

The planned data analysis is described for each of the main research questions below. For all analyses significance is set at *α* = 0.05, two-tailed tests.

#### 2.4.1 Data analysis research question 1

The hypotheses related to research question 1 addressing whether age is associated to both mean values and IIV of sleep parameters will be tested using location-scale mixed models. This model-based approach does not involve the calculation of traditional sleep variability metrics that are commonly used in sleep research (Fischer et al., 2021). By using this approach, we can directly probe the hypothesis that age affects both the mean and IIV of sleep. Another advantage of this analysis method is that time-varying covariates (e.g., weekend/weekday) can be included as predictors. Separate models will be run for the outcomes night sleep duration (actigraphy), sleep efficiency (actigraphy), bed times (actigraphy), rise times (actigraphy), mid sleep (actigraphy), and self-reported sleep quality. Location-scale mixed models allow to simultaneously model the mean as well as the within-person and between-person variation of the outcome variable as a function of the covariates (Hedeker et al., 2012). We here are particularly interested in the effect of age on the mean levels and within-person variation. To investigate whether the effect of age on the mean, respectively, IIV of the sleep measures is affected by covariates such as diurnal type [measured using the Diurnal type scale (Torsvall and Akerstedt, 1980)], insomnia [measured using the Insomnia Severity Index (Morin et al., 2011)], anxiety and depression [measured using the Hospital Anxiety and Depression Scale (Zigmond and Snaith, 1983)] and work status/retirement the models will be stepwise adjusted for covariates.

To investigate the secondary hypotheses, an interaction term between weekend vs. weekday sleep and age group will be included in the model. We plan to use the program MixWild (Dzubur et al., 2020).

#### 2.4.2 Data analysis research question 2

Research question 2 addressing whether sleep and adult age are associated to next-day cognitive performance will be evaluated using multilevel linear mixed models. Multilevel linear mixed models account for the nested structure of the data.

The primary outcome here is average number of correct trials per day. The secondary outcome is average percentage of correct trials per day. Main predictors of interest here are age group and sleep duration (H2.1), sleep efficiency (H2.2) and sleep quality (H2.3).

In order to disaggregate between- and within-person effects we will use person-mean centering of the time varying predictor variable (Hoffman and Stawski, 2009; Wang and Maxwell, 2015). Specifically, the time-varying predictor (e.g., sleep duration) will be person-mean centered resulting in 1) time-invariant predictor (level 2) that contains the person-specific mean and 2) a time-varying predictor (level 1), that is calculated by subtracting the person mean from the participant’s original covariate. The time-invariant person mean represents the between-person variation. The time-varying predictor captures within-person effects, e.g., whether sleeping 1 hour longer than usual is associated to a change in cognitive performance.

In order to test whether age moderates the effect of sleep on cognitive performance we will fit two competing models that will be compared using maximum likelihood testing. Model 1 will include main effects for both the sleep covariates (time invariant predictor and time varying predictor) and age group, but no interaction term. Model 2 will include both the main effects as model 1 and in addition the interaction between the sleep predictor and age group. The best fitting model will then be re-fitted with restricted maximum likelihood. Models will include a random intercept, and we will test whether including a random slope improves the model fit. We will also test whether an autoregressive (AR1) residual structure will improve the model fit. Analyses will be adjusted for relevant covariates. Data and residuals will be inspected in order to determine whether any transformation of data is needed. The data analysis for this research question will be carried out using the STATA procedure mixed.

#### 2.4.3 Data analysis research question 3

Research question 3 with hypothesis 3.1 to 3.6 that address whether daily variations in sleep and age are associated to both averages and IIV of positive and negative affect will be tested using mixed-effects location scale models.

The outcomes are positive affect, respectively, negative affect.

The main predictors here are age group and the time varying sleep variables (sleep duration, sleep efficiency, sleep quality). Time varying predictors (e.g., sleep duration) will be disaggregated into their between-subject and within-subject components. Analyses will be adjusted for relevant covariates.

To test whether age moderates the association between the sleep predictor and the outcome, we will fit two models, one which does include only main effects of the sleep predictor and age group, and one that includes in addition the interaction between age group and the sleep predictor. To probe the hypothesis, the effect of age group, the respective sleep predictor and potentially of the interaction of age group x sleep predictor on the mean levels and within subjective variance is of main interest. Models will be compared by using likelihood ratio tests, see, e.g., Nordgren et al. (2020). We plan to use the program MixWild (Dzubur et al., 2020) for the mixed effects location scale model.

##### Rationale on sample size

We plan to include *n* = 160 in the young adult group and *n* = 160 in the older adult group, resulting in a total *n* = 320 participants. The sample size, together with the 21 day duration of the protocol and the number of daily measures result in a large data set compared to the majority of previous studies that address the relationship between daily variations in sleep and cognitive performance (Lücke et al., 2022) and mood [see, e.g., review by Konjarski et al. (2018)].

Conducting a formal *a priori* sample size calculation is considered the best way to determine sample size, but proved challenging in the current context. For this type of intensive longitudinal data, simulation based approaches are considered the best option to conduct power analysis (Lafit et al., 2021). Yet, to perform the Monte Carlo simulations, the researchers need to have prior detailed knowledge on which results to expect in order to be able to enter those parameters in the *a priori* power analysis. Unfortunately, we here lack knowledge for many of the required input variables. Of note, data from small pilot studies should not be used as input for sample size calculations either as the results are not reliable due to small sample size (Albers and Lakens, 2018).

In order to have some indication on a sufficient sample size, we here used a combination of input parameters that are based on previous research together with values that we considered to be reasonable estimates to perform a power analysis for H2.1b, focusing on the cross-level interaction effect between the within-person component of sleep duration and age group. Please note, that due to the uncertainty on the input variables, the results of the power analysis need to be interpreted with caution. We chose to focus on the interaction term rather than main effects in order to obtain a more conservative estimate.

We used model 6 [*Group differences in the effect of a Level 1 continuous predictor (fixed slope)*] of the R shiny app PowerAnalysisIL (Lafit et al., 2021) to perform a power analysis, which is a simplified model compared to the planned analysis. Here we assumed that 1 h longer sleep than usual would result in an increase of 0.8 correct responses in the mDSST, respectively, 1 h shorter sleep would result in a decrease of 0.8 correct responses in the mDSST. We assumed that this effect would be attenuated by 1/3 (i.e., −0.27) in the older age group. Information on other input values used for the power analysis can be found in the Supplementary material. The results of the power analysis indicated that a sample size of *n* = 160 in each group would be sufficient to reach 86% power for the cross-level interaction effect between age group and the within-person component of sleep duration.

### 2.5 Compliance, missing data and data exclusion

A recent analysis of a pooled data set found a compliance rate of 78% in ESM studies using 10 daily prompts (Rintala et al., 2019). The assessment period in this study is with 21 days longer than the studies included in this analysis and study length was associated to a slight decrease in compliance (Rintala et al., 2019). In order to ensure compliance, we employ a number of strategies: Firstly, participants are individually briefed in person and called up after about 2–3 days to check in (Palmier-Claus et al., 2011). Participants are also welcome to reach out to the study assistants in case of questions during the data collection. Secondly, we use a staggered compensation scheme depending on the contribution of the participants. According to a meta-analysis, incentives are associated to increased compliance (Vachon et al., 2019). Thirdly, the participants are given access to a visualization of parts of their own data at the end of their respective data collection. Fourthly, we only use a limited number of questions per ESM questionnaire (< 30 questions), as previous research showed that a short length of the questionnaire (30 vs. 60) is more important than the frequency of notifications when it comes to both the experience of the participants and the quality of answers (Eisele et al., 2022).

After the data collection is completed, individual data might be excluded from the analysis due to either the interpretability of the data being compromised (e.g., technical dysfunction of the phone, significant life event) or visualization of data, respectively, residuals suggesting that a datapoint is an outlier / influential observation. Exclusion of data and/or transformation of data will be made transparent in publications. While it is a common practice in many ESM studies to include a minimum required response frequency (e.g., 1/3 of the prompts), these thresholds are arbitrary (Viechtbauer, 2022). Thus, we here do not plan to use thresholds *per se*, but only in conjunction with sensitivity analyses.

## 3 Discussion

The project’s purpose is to contribute to the current knowledge on how day-to-day variations in sleep are related to performance and affect in the everyday life of younger and older adults. We here combine mobile ESM that encompasses both self-reports and cognitive testing with actigraphy over a period of 21 days.

This project will make several contributions to the research front. Firstly, it will contribute to the understanding of how adult age is related to mean values and intra-individual variability of sleep duration, sleep timing and sleep quality in everyday life.

Secondly, the project will provide new knowledge on how daily variations in sleep are related to next-day cognitive performance. While it is well-known that sleep deprivation impairs cognitive performance, there is a surprising lack of knowledge on whether sleeping shorter or longer than usual is associated to changes in objectively measured performance in the context of everyday life. Moving the cognitive testing outside the strictly controlled laboratory environment is a promising complementary avenue (Moore et al., 2017). Testing on a single day in the laboratory that may include either requirements imposed by the researcher (e.g., no caffeine while the individual usually drinks coffee) or preparatory actions of the participant themselves (e.g., go to bed earlier than usual), may not always be representative of their performance during a regular day. Moreover, repeated testing of cognitive functioning across several days, which is due to high costs usually not possible in a laboratory environment, can benefit a better understanding on how variable cognitive performance is and the factors determining this variability (Moore et al., 2017). Conducting cognitive testing in individual’s regular environment may also prevent adverse and unwanted influences of contextual effects of the laboratory environment. For instance, it has previously been shown that the traditional laboratory environment affects older adults differently than younger adults (Sindi et al., 2013).

Thirdly, the results of the project will benefit new insights on the link between sleep and affective functioning, as we will here investigate the association of sleep not only with mean affect but also with affect variability. Affect variability is associated to poorer health, above and beyond mean levels of affect (Jenkins et al., 2020). A better understanding on whether day-to-day changes in sleep have a direct relationship with next day affect can for instance inform new treatment approaches.

Fourthly, the project will greatly benefit the knowledge on whether and how the role of sleep for performance and affective well-being changes across the life span. So far, little is known on whether the age differences found in experimental sleep deprivation studies can also be observed in everyday life. Here, we therefore directly address whether adult age moderates the associations between daily variations in sleep and next-day performance and affect.

Apart from addressing these research gaps, the project has several methodological strengths. With a duration of 21 days, the ESM period is longer than many previous studies. Including 3 weekends per participant will help us to better estimate any weekend / weekday differences, which are particularly interesting from an aging perspective (Åkerstedt et al., 2019). Also, the number of daily questionnaires with 8 semi-randomly prompted daytime questionnaires and additional morning and evening questionnaire is higher than what most previous studies in the sleep field used, which will allow a better understanding of the fluctuations during the day. We opted here to include several daily questionnaires, as variables of interest likely fluctuate during the day. There seems so far to be little systematic knowledge on which number of daily assessments offers a good balance between participant burden and information gain, and traditions in different research fields seem to vary. As indication, studies in the sleep field usually use rather few and/or aggregated daily assessments of affect [for an overview of studies on sleep and daily mood, see, e.g., review by Konjarski et al. (2018)], while ESM studies in depressive patients frequently use even 10 questionnaires per day (Telford et al., 2012; Wichers et al., 2012; Hartmann et al., 2015). Apart from the higher number of daily questionnaires, and the longer data collection period, the number of participants here is relatively large compared to many previous ESM studies of sleep, resulting in an overall large data set.

A further advantage here is that we use mobile technology to deliver the daily questionnaires which ensures that answers reflect the momentary experiences and minimizes the risk for retrospective bias. A further strength is that we include actigraphy as an objective indicator of sleep duration and efficiency. Self-reported sleep does often not match well with objective indicators (Jackowska et al., 2011; Aili et al., 2017). Moreover, both age (Akerstedt et al., 2016) and insomnia (Harvey and Tang, 2012) seem to be related to systematic bias in self-reported sleep, further stressing the importance of including objective indicators.

### Limitations

In this study we use a combination of different measurements, namely self-reports, cognitive performance tests and actigraphy with each of them having their respective limitations and strengths. A common criticism of self-report data is that they require participants to be aware of their thoughts, feelings and behaviors and truthfully report them, and that self-report data are by nature subjective. Yet, self-report measures allow insights in the state of the participant that cannot be gained otherwise. Moreover, during recent years self-report measures are increasingly shown to actually be linked and predictive of traditional objective measures. For instance, self-reported hedonic well-being is not only bidirectionally linked with health but may even prospectively predict health outcomes in older age (Steptoe et al., 2015). Likewise, the KSS as measure of subjective sleepiness, that we also use in this study, has been shown to be a sensitive and valid indicator of insufficient sleep and impaired waking function (Akerstedt et al., 2014). Cognitive testing via mobile phone app: While the use of a performance test clearly is a strength of this study, and mobile cognitive testing is a promising area, there are certainly also drawbacks of this method (Moore et al., 2017). Differences between the hardware and software and mobile phones may influence the performance on the cognitive tasks. However, here the primary focus of the analysis is on studying intra-individual changes in performance. Moreover, cognitive testing in daily life situations may suffer from interference from the environment. Actigraphy is generally considered a valid and reliable way to objectively measure sleep and wakefulness, but is still less accurate than the gold-standard polysomnography (Sadeh, 2011). Also, actigraphy cannot be used to deduce any information regarding sleep stages.

While we here apply inclusion and exclusion criteria that are less strict than most sleep deprivation studies which hopefully results in a sample that is more representative to the general population, it still needs to be considered that the included sample may differ from the general population as we do not use random sampling from the population and self-selection effects are in play.

### Conclusion

The current project utilizes mobile ESM in combination with objective, actigraphic sleep recording to increase the knowledge of day-to-day sleep variability and the pivotal role of sleep for cognitive performance and affective well-being in young and older adults’ everyday life.

## Ethics statement

This study was approved by the Swedish Ethical Review Authority (2022-04683-02). Written informed consent is obtained from all participants.

## Author contributions

JS: Conceptualization, Funding acquisition, Writing – original draft. MF: Writing – review & editing. WL: Writing – review & editing. TÅ: Conceptualization, Funding acquisition, Writing – review & editing. GK: Conceptualization, Funding acquisition, Writing – review & editing.

## Footnotes

^1^ https://osf.io/8sg6v/

^2^ www.psymate.eu
