# Supplementary material for: Sleep in everyday life – relationship to mood and performance in young and older adults: a study protocol
Source: Front Psychol. 2023 Nov 23;14:1264881. doi: 10.3389/fpsyg.2023.1264881 (PMC10701737; doi:10.3389/fpsyg.2023.1264881)
Supplement: Supplementary file 1 [file Data_Sheet_1.PDF]

Sleep in everyday life – relationship to mood and performance in young and older adults: a study protocol

Johanna Schwarz[1,2], Malin Freidle[3], Wessel van Leeuwen[1], Torbjörn Åkerstedt[1,2], Göran Kecklund[1,2]

[1] Stress Research Institute, Department of Psychology, Stockholm University, Stockholm, Sweden
[2] Department of Clinical Neuroscience, Karolinska Institute, Stockholm, Sweden
[3] Department of Psychology, Stockholm University, Stockholm, Sweden

Supplementary material 1

Input values power analysis

The fixed intercept β_{00} is set to 24: This estimate is based upon published data of a study using the 30s version of the mDSST(Verhagen et al., 2019). In the age group of participants <=30 years (n=26) the mean number of correct trials was 11.6 for the 30s version.

The effect of the level-2 dummy variable on the intercept β_{01} is set to -3. While there is a lack of previous studies on age differences in performance in mobile versions of the DSST, it is well-established that older adults are performing worse on the DSST (Hoyer et al., 2004).

The fixed slope is set to 0.8; i.e. we expect that the young adult participants have 0.8 more correct responses when sleeping 1 hour more than their average sleep duration.
Van Dongen et al. (2003) used a computerised 1.5min digit symbol substitution task comparing sleep restriction to 8 hours time in bed. According to the figure published in the article, the performance in the 6h sleep group was about 3 items less on day 1 of the sleep restriction protocol compared to the 8h sleep group.

The effect of the level-2 dummy variable on the slope is set to -0.27, meaning that the effect of the sleeping 1 hour more than average sleep duration is reduced by about 1/3 compared to young adults. To our knowledge there are no previous studies addressing adult age difference in DSST performance after sleep deprivation or in relation to daily changes in sleep duration, but performance deficits are generally attenuated in older adults after sleep loss.

The standard deviation of level-1 errors ($\sigma\epsilon$) is set to 4. The autocorrelation of level-1 errors ($\rho\epsilon$) is set to 0.3. The standard deviation of random intercept ($\sigma v0$) is set to 4.

Input values for mean and standard deviation for sleep duration are derived from an analysis of pooled data on sleep variability (Messman et al., 2022), using the values for actigraphy sleep duration (mean and iSD) in the age groups 18-24 years respectively 55-64 years as reference. Specifically, the mean of time-varying variable X in Group 0 is set to 6.85h. The standard deviation of time-varying variable X in Group 0 is set to 1.28h. The mean of time-varying variable X in Group 1 is set to 6.77h. The standard deviation of time-varying variable X in Group 1 is set to 0.88h.

Hoyer, W.J., Stawski, R.S., Wasylyshyn, C., and Verhaeghen, P. (2004). Adult age and digit symbol substitution performance: a meta-analysis. *Psychol Aging* 19(1)**,** 211-214. doi: 10.1037/0882-7974.19.1.211.

Messman, B.A., Wiley, J.F., Yap, Y., Tung, Y.C., Almeida, I.M., Dietch, J.R., et al. (2022). How much does sleep vary from night-to-night? A quantitative summary of intraindividual variability in sleep by age, gender, and racial/ethnic identity across eight-pooled datasets. *J Sleep Res* 31(6)**,** e13680. doi: 10.1111/jsr.13680.

Van Dongen, H.P., Maislin, G., Mullington, J.M., and Dinges, D.F. (2003). The cumulative cost of additional wakefulness: dose-response effects on neurobehavioral functions and sleep physiology from chronic sleep restriction and total sleep deprivation. *Sleep* 26(2)**,** 117-126.

Verhagen, S.J.W., Daniëls, N.E.M., Bartels, S.L., Tans, S., Borkelmans, K.W.H., de Vugt, M.E., et al. (2019). Measuring within-day cognitive performance using the experience sampling method: A pilot study in a healthy population. *PLoS One* 14(12)**,** e0226409. doi: 10.1371/journal.pone.0226409.
